# Metabolic Variations in Brown Rice Fertilised with Different Levels of Nitrogen

**DOI:** 10.3390/foods11213539

**Published:** 2022-11-07

**Authors:** Yichao Ma, Shuang Zhang, Zhaoxia Wu, Wentao Sun

**Affiliations:** 1 College of Food Science, Shenyang Agricultural University, 120 Dongling Rd., Shenyang 110866, China; 2 Institute of Plant Nutrition and Environment Resources, Liaoning Academy of Agricultural Sciences, 84 Dongling Rd., Shenyang 110000, China

**Keywords:** brown rice, metabolism, antioxidant activity, nitrogen fertiliser

## Abstract

Nitrogen is a necessary element for plant growth; therefore, it is important to study the influence of N fertilisers on crop metabolites. In this study, we investigate the variability of endogenous metabolites in brown rice fertilised with different amounts of nitrogen. We identified 489 metabolites in brown rice. Compared to non-nitrogen fertilised groups, there were 59 differentially activated metabolic pathways in the nitrogen-fertilised groups. Additionally, there were significantly differential secondary metabolites, especially flavonoids, between groups treated with moderate (210 kg N/hm^2^) and excessive amounts of nitrogen (420 kg N/hm^2^). Nitrogen fertilisation upregulated linoleic acid metabolism and most steroids, steroid derivatives, and flavonoid compounds, which have antioxidant activity. The DPPH, ABTS, and hydroxyl radical scavenging rates were higher in fertilised groups than in the non-fertilised group. These findings provide a theoretical basis to enhance the health benefits of brown rice by improving fertilisation.

## 1. Introduction

Nitrogen is an essential nutrient for plant growth that affects external morphology and internal metabolic changes in plants [1]. Nitrogen metabolites provide primary materials for plant cell structure assembly, energy supply, physiological and biochemical reactions, and signalling. It is also an important component of various key cellular molecules such as proteins, nucleic acids, chlorophyll, and secondary metabolites, which are important for maintaining crop metabolism, growth, and stable yields. According to previous reports, agronomic treatment factors such as weather, temperature, and fertilisation can strongly influence grain metabolism and changes in metabolite abundance [2,3]. Fertiliser application is a convenient and effective way to control some of the environmental factors. Therefore, it is necessary to study the influence of the amount of nitrogen fertilisers on crop metabolites.

A recent epidemiological study has indicated that the intake of whole-grain products is associated with a reduced risk of various chronic diseases [4]. Brown rice, as a whole grain, is rich in high levels of biological activity and potentially beneficial health effects compared to white rice, while also providing more bioactive components with antioxidant activity, such as polyphenols and flavonoids, which have benefits for humans [5,6]. Most current studies on brown rice are investigating the effects of a single type of compound with functional activity and the antioxidative capacity of brown rice through phytochemical analysis. However, less attention has been paid to studying the impacts of nitrogen fertilisation on the metabolites of brown rice.

Metabolomics has received extensive attention in the field of grain research and has led to a series of important advances, especially in the regulatory mechanisms of traditional agricultural production [7]. In recent years, many researchers have applied metabolomics to study the mechanisms of the response of rice to environmental factors (drought, temperature), and the mechanisms of changes in rice chalkiness and yellowing [8]. Nevertheless, although some specific metabolite functions have been elucidated, studies on the key metabolites and metabolic pathways affected by nitrogen concentration in brown rice are still limited. Since nitrogen fertilisation may have a multifaceted effect on metabolites in mature brown rice, effective metabolite detection methods will help to understand the effect of nitrogen fertilisation on brown rice. Therefore, it is necessary to study the differences in metabolites in brown rice fertilised with different amounts of nitrogen using metabolomics and establish a metabolic network.

In this study, we selected a local variety of brown rice, Yanfeng 47, for the study of agricultural production in the Panjin region of Liaoning, China. We used metabolomics analysis to study on the effect of nitrogen fertilisation on the accumulation of metabolic substances in brown rice. This study may help improve the nutritional value of edible brown rice by suggesting the application of an appropriate amount of fertiliser in agricultural production.

## 2. Materials and Methods

### 2.1. Rice Samples

The experiment area of the present study was located in the Liaohe Delta, Liaoning Province, China (122°14′16″ N, 41°9′30″ E). The local planting conditions in growing season are: average temperature in 2020 pf −4.9 °C, average annual precipitation of 30.8 mm, and average sunshine of 561.5 h. The seedlings were transplanted on 25 May and were harvested on 8 October in 2020. This sample used the local main cultivar, Yanfeng 47. Indicators of experimental soils are shown in Table 1. Based on a previous study (Liang et al., 2021), the conditions for nitrogen application (0, 210 and 420 kg N/hm^2^) were set. The dispersion was a control group (N0) with non-nitrogen treatment and two treatment groups (N2 and N6) treated with nitrogen (210 and 420 kg N/hm^2^, respectively). The experiment was conducted using six biological replicates.

### 2.2. Sample Preparation and Extraction for Non-Targeted Metabolomics Analysis

After freeze-drying for 48 h, the sample was ground for 2 min at 60 Hz. Then, two hundred micrograms were taken and dissolved by 70% aqueous methanol. The mixture was stored at 4 °C and extracted by six one-minute cyclones. After, the samples were centrifuged the supernatant was collected. Then, it was filtrated through 0.22 μm and transferred into vials. Six replicates were made for each variety.

### 2.3. Antioxidant Activity Assay

#### 2.3.1. Determination of DPPH Radical Scavenging Activity

The method in [9] was used with slight modifications to assess the DPPH• scavenging activity of each extract. The mixtures were shaken vigorously and the sample represented by Ai was incubated for 30 min in the dark. Absorbance of solution was measured at 517 nm. The calculation formula is as follows:

$$\mathrm{scavenging}\;\mathrm{effect}\;\left(\%\right)=1-\frac{A_{sample}-A_{background}}{A_{control}}\times 100\%$$

where *A_sample_*, *A_control_*, and *A_background_* refer to the absorbance of the sample solution (sample and DPPH), control solution (without sample), and background solution (without sample), respectively.

#### 2.3.2. Determination of Hydroxyl Radical Scavenging Activity

Each extract was assessed as previously described in [10] with slight modifications. 1 mL of anhydrous ethanol solution (with 1.865 mmol/L phenanthroline monohydrate) and 1 mL phosphate buffer (pH 7) were added to 2 mL of the samples. The mixtures were then vigorously shaken, and 1.0 mL of 1.865 mmol/L FeSO_4_ solution was added. Then, 1.0 mL of 0.03% H_2_O_2_ was added to water at 37 °C for 60 min. The absorbance was measured at 536 nm. The hydroxyl radicals were obtained via interpolation using linear regression analysis.

$$\mathrm{scavenging}\;\mathrm{effect}\;\left(\%\right)=\frac{A_{S}-A_{n}}{A_{b}-A_{n}}\times 100\%$$

where *A_n_*, *A_s_*, and *A_b_* refer to the absorbance of the control solution (water replaces H_2_O_2_), sample solution, and background solution (without sample), respectively.

### 2.4. KEGG Pathway-Enrichment Analysis

To analyse the differential metabolites, metabolic pathways were investigated using http://www.genome.jp/kegg/pathway.html (2022). The metabolites identified were then observed for the change level in the KEGG pathway. Significantly enriched pathways were determined by both a hypergeometric test and a threshold *p*-value < 0.05.

### 2.5. Data Analysis

Raw data were processed into common data format (mzML) files. The robust LOESS signal correction (QC-RLSC) was applied for data normalisation to correct for any systematic bias. After normalisation, only ion peaks with relative standard deviations (RSDs) less than 30% in QC were kept to ensure proper metabolite identification. In the multivariate statistical analysis, principal component analysis (PCA) was used to compare metabolic profiles. Orthogonal-projections-to-latent-structures discriminant analysis (OPLS-DA) was performed to discriminate between different groups. All the models evaluated were tested for overfitting with permutation tests. The descriptive performance of the models was determined by R^2^X (cumulative) (perfect model: R^2^X (cum) = 1) and R^2^Y (cumulative) (perfect model: R^2^Y (cum) = 1) values, while their prediction performance was measured by Q^2^ (cumulative) (perfect model: Q^2^ (cum) = 1) and a permutation test. The permuted model should not be able to predict classes; R^2^ and Q^2^ values at the *Y*-axis intercept must be lower than those of Q^2^ and the R^2^ of the non-permuted model. In parallel, the metabolites identified were validated at a univariate level using FC > 2 or FC < 0.5 [11].

## 3. Results

### 3.1. Non-Targeted Metabolomics Analysis in Brown Rice

**Figure 1:**
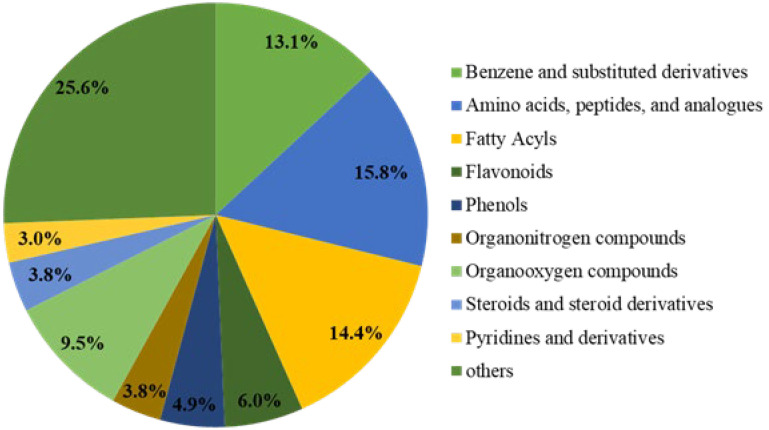
Pie chart depicting the biochemical categories of the differential metabolites.

To clearly understand the differences in the chemical composition of brown rice under three nitrogen fertilisation amounts, we used non-targeted metabolomics analysis to identify metabolites among three groups of brown rice samples with different levels of nitrogen fertilisation. The three groups were the following: control (N0) without nitrogen treatment (0 kg N/hm^2^) and two treatment groups (N2 and N6) treated with nitrogen (210 and 420 kg N/hm^2^, respectively). The changes of the primary metabolites in the three groups of brown rice with different nitrogen fertiliser rates are shown in Figure 1. A total of 10,946 primary metabolites were identified in N0 vs. N2 vs. N6. Compared to N0, 1843 primary metabolites were upregulated and 911 metabolites were downregulated in N2, whereas 2695 primary metabolites were upregulated and 1355 were downregulated in N6. In total, 490 secondary metabolites were detected in all samples. As shown in Figure 1, these were categorised into ten classes. Moreover, secondary key metabolites (flavonoids, phenols, steroids, and steroid derivatives) were found to be related to the antioxidative property of brown rice.

### 3.2. Multivariate Analysis of Differentially Expressed Metabolites

**Figure 2:**
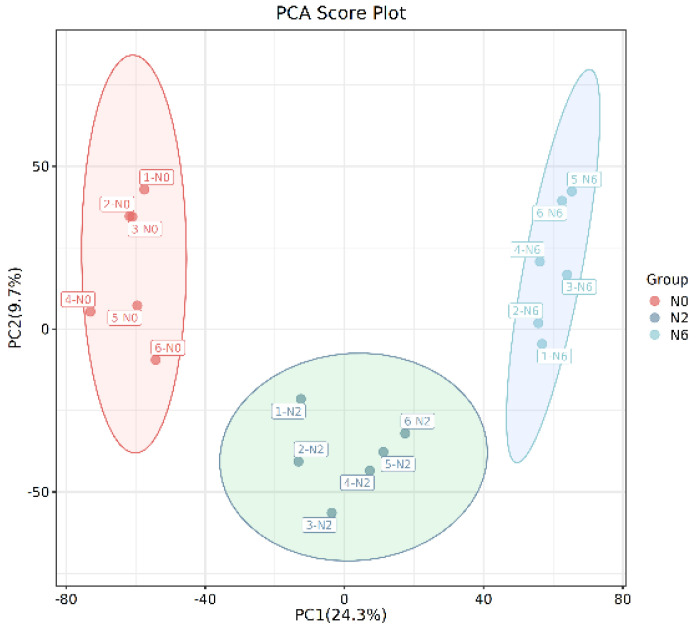
The PCA score plot.

Multivariate analysis methods are required to accurately determine differential metabolites because of the large volume and high-dimensionality of the metabolomics data. PCA was performed to analyse the identified metabolites using an unsupervised model (Figure 2A). The results revealed that the three groups’ samples were separated and divided into three different districts, suggesting that the groups had distinct metabolite profiles. Furthermore, PC1 and PC2 imply that the amount of nitrogen fertiliser remarkably impacts the metabolites in brown rice. The parameters and score-scatter plots of PLS-DA are presented in Table 2, revealing that the model was reliable and the three groups were well separated. Q^2^ was higher than 0.5. More importantly, the separation between non-nitrogenous brown rice and nitrogenous brown rice was notable.

Furthermore, the supervised OPLS-DA model was applied for pairwise comparison of metabolic characteristics. The results showed a significant difference among the three groups. Moreover, both the model interpretation rate R_2_Y (0.998) and the prediction rate Q_2_ (0.88) were high, confirming the good quality of each model.

### 3.3. Metabolomic Changes of Brown Rice with Different Nitrogen Amounts

**Figure 3:**
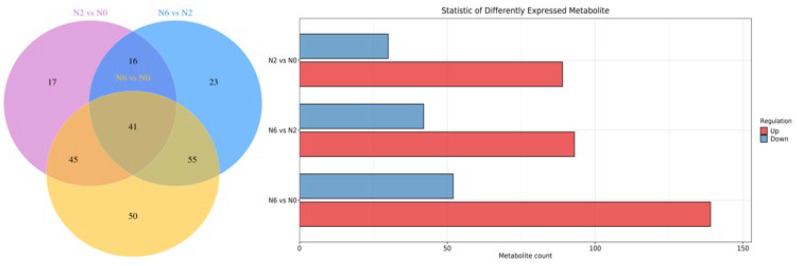

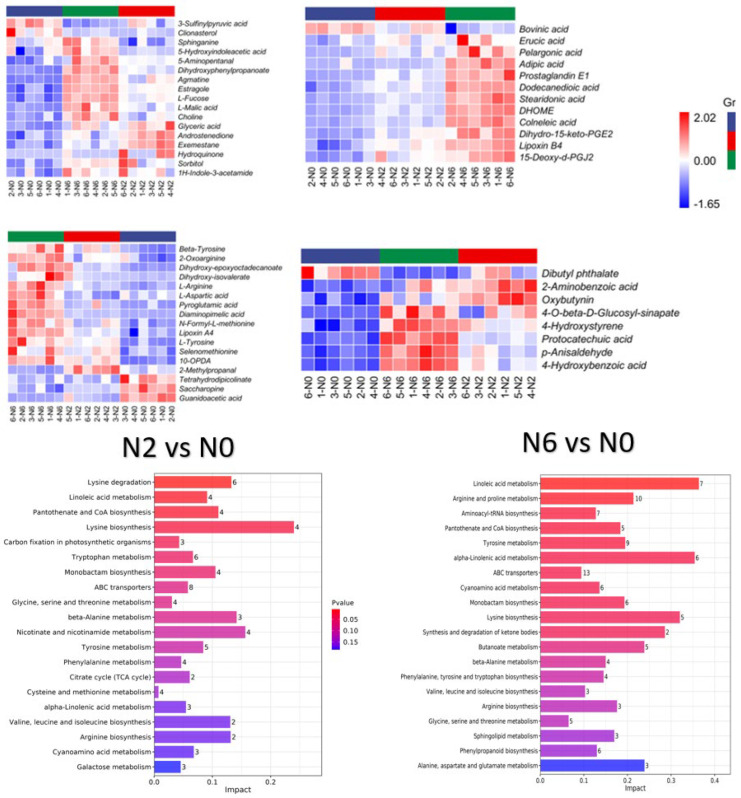
Venn diagram and statistics of differently expressed metabolite, KEGG enrichment bar chart.

In combination with OPLS-DA model analysis (VIP > 1.0), fold changes >2 or <0.5 were used as screening thresholds for detecting significantly differential metabolites among the three groups. As shown in the Venn diagram (Figure 3), 41 metabolites were detected in the three sample groups. We found 119 and 191 significantly differential metabolites between N2 vs. N0 and N6 vs. N0, respectively. Compared to N0, 89 metabolites were upregulated and 30 metabolites were downregulated in N2, whereas 139 metabolites were upregulated and 52 were downregulated in N6. Notably, compared to N0, the majority of amino acid metabolites were upregulated in N2 and N6. In particular, some key secondary metabolites in each pairwise comparison were involved in primary and secondary metabolism, suggesting the high nutritional value of brown rice.

The grouped clustering heat map (Figure 3) showed that nitrogen fertilisation increased the content of most substances in the category of benzene and substituted derivatives in brown rice. However, dibutyl phthalate content decreased with increasing nitrogen fertilisation. The contents of benzene and substituted derivatives changed significantly in N6 compared to N2. Carboxylic acids and their derivatives are involved in glycolysis, the pentose phosphate pathway, and the tricarboxylic acid cycle in plants, providing energy and acting as synthetic precursors for secondary metabolism. The content of carboxylic acids and derivatives in the N2 and N6 groups significantly differed (*p* < 0.05) compared to N0 (Figure 3). Tetrahydrodipicolinate, saccharopine, guanidoacetic acid, and the three carboxylic acids and derivatives decreased with increasing nitrogen fertilisation content. The levels of the remaining metabolites showed an increasing trend. Notably, the relative content of 2-methylpropionaldehyde was higher in N2 than in N0. Additionally, the fatty acid content differed significantly among the three groups, especially in the N6 group, which had significantly increased levels of all substances except for bovinic acid. Some secondary metabolites such as organooxygen compounds and organonitrogen compounds, were also significantly increased, probably because of the increase in N content in plants due to N fertilisation. Moreover, there were also differences in some steroids and steroid derivatives, including phenylpropanoic acids, keto acids and derivatives, and flavonoid compounds with antioxidant activity. Among them, 3-sulfinylpyruvic acid and clionasterol were decreased under high-nitrogen conditions. Collectively, these results indicate that the content of metabolites in brown rice varies with the content of nitrogen in fertilisers.

### 3.4. KEGG Classification and Enrichment Analysis

The results of metabolic pathway enrichment are shown in Figure 3. KEGG classification revealed 62 metabolic pathways in N2 vs. N0, and 68 metabolic pathways in N6 vs. N0. These pathways were classified as “metabolism”, including amino acid metabolism, biosynthesis of other secondary metabolites, carbohydrate metabolism, energy metabolism, lipid metabolism, metabolism of cofactors and vitamins, metabolism of terpenoids and polyketides, and nucleotide metabolism. Two differentially activated pathways (ABC transporters and aminoacyl-tRNA biosynthesis) were classified as environmental information processing and genetic information processing. Additionally, lysine biosynthesis and linoleic acid metabolism were significantly enriched in N2 and N6 compared to N0.

### 3.5. Antioxidant Activity

**Figure 4:**
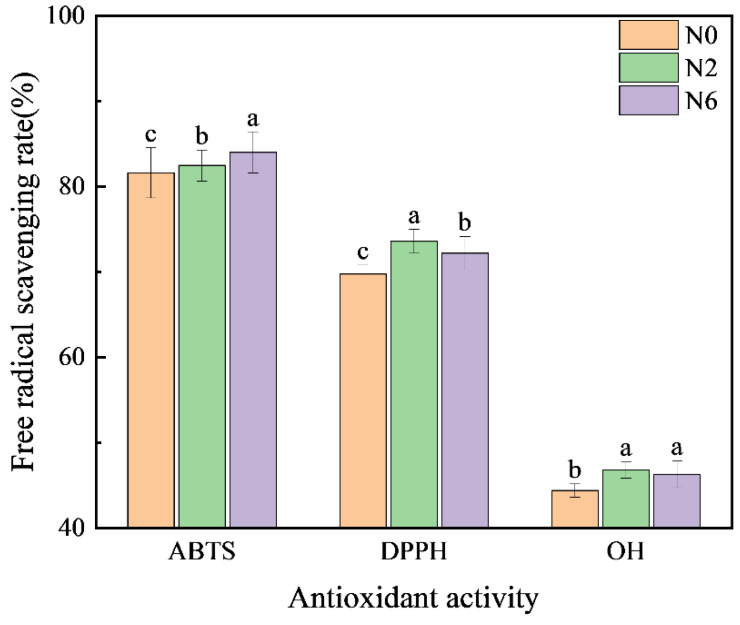
Antioxidant activity of brown rice at different amounts of nitrogen fertilisation. Values of each bar with no letters in common are significantly different (*p* < 0.05).

Several secondary metabolites in brown rice exert antioxidative activities via various mechanisms, including prevention of chain initiation, peroxide decomposition, the combination of transition metal ion catalysts, reduction of free radical absorption capacity and scavenging ability, and prevention of continuous hydrogen extraction [12]. Therefore, to fully analyse the antioxidative capacity of rice, we used the following three methods (Figure 4): ABTS methods, which are based on single-electron donation; DPPH methods, which are based on providing hydrogen; and hydroxyl free scavenging. The antioxidative activity of N2 and N6 was higher than that of N0. However, DPPH and hydroxyl scavenging activities in N6 were lower than in N2. This may be due to the significant differences in metabolites with antioxidative activity between the two groups. Therefore, the different antioxidative capacity of brown rice is probably due to interactions between different metabolites, and nitrogen fertilisation improves the antioxidative activity.

### 3.6. Enzymatic Activity

**Figure 5:**
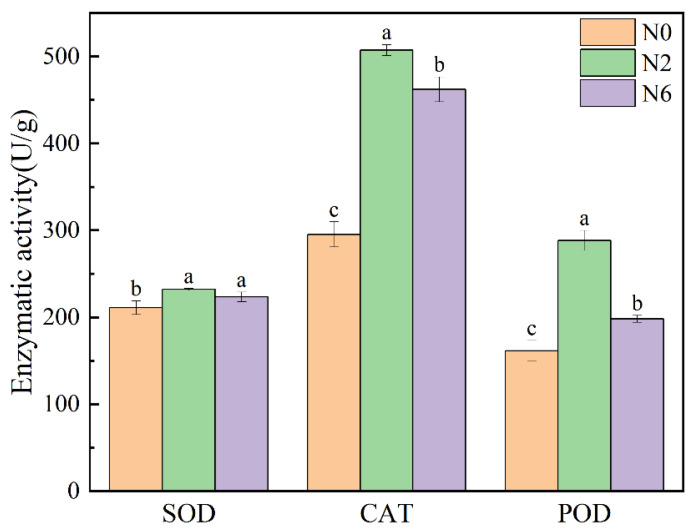
Enzymatic activity of brown rice at different amounts of nitrogen fertilization. Values of each bar with no letters in common are significantly different (*p* < 0.05).

Plants have an effective antioxidant defence system, including the enzymatic system, which can affect the level of antioxidant activity. Superoxide dismutase (SOD) can catalyse superoxide radical (O^2−^) disproportionation. Catalase (CAT) is a kind of antioxidant enzyme that exists in living organisms. Peroxidase (POD) uses hydrogen peroxide as electron acceptor to catalyse substrate oxidation. These two enzymes can reflect the antioxidant activity in rice. Therefore, this experiment measured the enzyme activity of SOD and POD in three groups under the different levels of nitrogen application (Figure 5). There were significant differences in SOD, CAT, and POD in brown rice with different levels of nitrogen fertiliser (*p* < 0.05). The SOD enzymatic activity of N2 and N6 is higher than that of N0. However, the CAT and POD activity of N2 was higher than that of N6 and N0. The two enzymatic activities of N2 were the highest, which was consistent with the results of antioxidant activity. SOD, CAT, and POD can improve the antioxidant capacity by catalysing the reaction of some secondary metabolites with antioxidant activity in plants. Therefore, moderate nitrogen fertiliser application can improve the antioxidant capacity of brown rice.

## 4. Discussion

**Figure 6:**
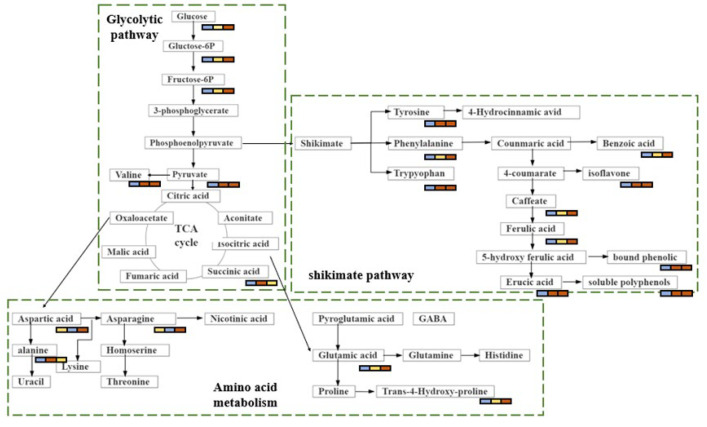
The metabolic changes associated with biochemical pathways.

Brown rice is currently becoming popularity among consumers because of its potential health benefits [4]. It is reported to contain many bioactive compounds, such as polyphenols, flavonoids, anthocyanins, tocopherols, and sitosterol [13]. Brown rice has been shown to have antioxidative, anti-inflammatory, antidiabetic, and anticancer properties, and it can also decrease the risk of metabolic diseases [14,15,16]. Nevertheless, little is known about the accumulation of secondary metabolites in brown rice. In this study, we show that the levels of nitrogen fertilisation can induce remarkable metabolic changes in brown rice. The identification of these metabolites may be useful for the development of potentially high-nutritional-value foods and may provide recommendations for the appropriate level of nitrogen fertilisation in agricultural production. The metabolic changes associated with biochemical pathways in response to various amounts of nitrogen are shown in Figure 6 to provide a clear description of the changes in metabolic regulation. Although we did not directly demonstrate the modulatory effect of these metabolites on the antioxidative activity of brown rice, comparative analysis revealed that these metabolites are mainly involved in two major metabolic pathways: carbohydrate metabolism and amino acid metabolism. Based on the analysis of the metabolic pathways, we concluded that one metabolic pattern may be critical in brown rice fertilised with nitrogen. The is the shikimate acid–phenylalanine-flavonoid mode. The precursors of this mode originate from the glycolytic and tricarboxylic acid cycle (TCA) pathways in carbohydrate metabolism.

### 4.1. Carbohydrate Metabolism

Changes in carbohydrates in a plant are directly related to plant growth [17]. L-fucose and sucrose levels were significantly higher in N2 and N6 than in N0. These sugars are mainly involved in sugar metabolism, which in turn affects glycolytic pathways, Additionally, sugar metabolism may also increase the level of phosphohydroxypyruvic acid, indirectly affecting the rate of the TCA cycle. Notably, 6-phosphogluconic acid, glyceric acid, and gluconolactone levels were higher in N2 than in N6. These compounds are derived from the phosphopentose pathway and are regulated by carbon metabolism, which is highly dependent on nitrogen assimilation. In contrast, nitrogen assimilation calls for a plentiful supply of energy and carbon skeleton from carbon metabolism [18]. In the high-nitrogen-fertilisation group (N6), nitrogen metabolism accelerates the energy consumption of carbon metabolism, which leads to lower levels of these metabolites in the phosphopentose pathway. In addition, the increased metabolism of carbohydrates was also reflected in other highly accumulated sugars and sugar derivatives, including α-lactose and galactonic acid. Carbon assimilation and nitrogen uptake regulate plant growth and development. Some studies have shown that carbon and nitrogen metabolism in amino acid, carbohydrate, and lipid metabolism are interdependent and interact with each other [19]. Therefore, high-nitrogen fertilisation improves the content of carboxylic acids and their derivatives to promote carbon metabolism.

### 4.2. Amino Acid Metabolism

Amino acid metabolism is closely related to glycolysis and the TCA cycle. In this study, the level of amino acid changes in N2 and N6 was statistically significant when compared with N0. The amino acid metabolic pathway is the main biological pathway for synthesis of flavonoids and polyamines. The important amino acids associated with their biosynthesis are aromatic amino acids (phenylalanine, tyrosine, and tryptophan) and aliphatic amino acids (ornithine and lysine) [20]. We observed that phenylalanine accumulation was higher in brown rice fertilised with nitrogen (N2 and N6) than in non-fertilised rice. Directly affecting the flavonoid biosynthesis pathway is the upstream phenylpropanoid biosynthesis pathway, and upstream substances are derived from cinnamic acid [21], which is formed from phenylalanine, by increasing the activity of phenylalanine deaminase [22]. There was significant change in cinnamic acid content in N2 and N6. However, the contents of phenylalanine and flavonoids increased as the amount of nitrogen fertiliser increased; therefore, the nitrogen fertiliser may have affected the activity of phenylalanine deaminase. In addition, the current study highlighted that compared to N0, lysine degradation, lysine biosynthesis, and tryptophan, tyrosine, and phenylalanine metabolism were significantly enriched in N2, whereas tyrosine, arginine, and proline metabolism and lysine biosynthesis were significantly enriched in N6. These metabolism-related amino acids, including lysine, tryptophan, tyrosine, phenylalanine, arginine, and proline, which are derived from the TCA cycle intermediate α-ketoglutaric acid, were all increased in brown rice fertilised with nitrogen. This is consistent with [20]. showing that these amino acids are associated with the biosynthesis of flavonoids. Research has indicated that the relative contents of lysine and phenylalanine increased in wheat grains after the addition of nitrogen [2].

The metabolic pathways of aliphatic amino acids also changed significantly with nitrogen fertilisation. In short, nitrogen-containing molecules (glutamate, glutamine, and asparagine) are involved in nitrogen assimilation, circulation, transport, and storage [23]. A previous study has shown that high-nitrogen fertiliser treatment accelerated the accumulation of these nitrogenous amino acids [24]. Hence, N6 is relatively high in most nitrogenous compounds.

### 4.3. Shikimate-Phenylpropanoid-Flavonoid Metabolism

Four major metabolic pathways are usually taken part in the synthesis of secondary metabolites in cereals: the C2 acetate pathway, C5 isovaleric acid pathway, C6-C3 phenylpropylene pathway, and amino acid pathway [25]. Polyphenolic compounds are usually produced via the C2 acetate pathway, whereas flavonoids are usually produced via the shikimate and acetate pathways [26]. In this study, most of the significantly differential pathways between N- and non-N-fertilised brown rice were classified as the shikimate pathway. The shikimate pathway provides carbon skeletons for the secondary metabolic pathways of plants, such as the aromatic amino acid metabolism and aromatic phytochemical (flavonoid) biosynthesis pathways [27]. This explains the significant changes in flavonoid levels in brown rice fertilised with different amounts of nitrogen. Additionally, flavonoids exhibit antioxidative activities; therefore, the antioxidative activity was also affected by the amount of nitrogen fertilisation. Phenylalanine, tryptophan, and tyrosine related to the shikimate pathway were increased in brown rice of nitrogen fertilisation compared with non-fertilised rice. There are many antioxidant-active substances in plants, such as polyphenols and flavonoids, whose precursors are aromatic amino acids and shikimic acid. These precursors undergo flavonoid biosynthesis through the shikimate pathway. They are able to scavenge or inhibit reactive oxygen species and thus achieve antioxidant functions that protect the body [28]. Aromatic amino acids are precursors of secondary metabolites, including cinnamic acid, 4-coumarate, caffeate, and 4-hydroxycinnamic acid [29]. In present research, the antioxidants benzoic acid and 4-hydroxycinnamic acid were upregulated. The upregulation of antioxidants could be a reaction to ROS overproduction due to high N content, consistent with the recent findings that the increase in defence-related metabolites derived from the shikimate-phenylpropanoid metabolism might be connected to high nitrogen levels [30]. Under low-nitrogen conditions, plants synthesise primary metabolites; however, under high-nitrogen conditions, plants often synthesise secondary metabolites [31]. This may explain the high flavonoid content in the N6 group.

## 5. Conclusions

Brown rice is rich in bioactive substances and has several health benefits. In this study, we indicated that nitrogen fertilisation increased the contents of flavonoids and the regulation of amino acids in the phenolpropane metabolic pathway, thereby increasing the synthesis of downstream compounds with antioxidant activity. Although the antioxidative activity in N2 and N6 was significantly higher than in N0 brown rice, the succinic acid content in N6 was lower than that in N2, but higher than that in N0, and the DPPH and hydroxyl radical-scavenging rates in N6 were slightly lower than those in N2, suggesting that high-nitrogen fertilisation does not contribute to antioxidant activity. Therefore, we suggest that moderate nitrogen fertilisation could appropriately increase the antioxidative activity of brown rice by regulating its metabolites. These findings can help in the agricultural production of rice and provide a theoretical basis for the development of functional edible brown rice with high nutritional value.

## Figures and Tables

**Table 1:** Indicators of experimental soils.

Soil pH	Organic Matter (mg/kg)	Total Nitrogen (g/kg)	Available Nutrient (mg/kg)			Bulk Density (g/cm^3^)
			N (Nitrogen)	P (Phosphorus)	K (Potassium)	
8.2	22.57	1.42	105.24	21.61	164.22	1.39

**Table 2:** The parameters of multivariate analysis of brown rice under different nitrogen levels.

Multivariate Analysis	Group	Positive Ion Model			Negative Ion Model		
		R2X	R2Y	Q2	R2X	R2Y	Q2
PLS-DA	N2 vs. N0	0.305	0.994	0.757	0.389	0.999	0.816
	N6 vs. N0	0.471	0.999	0.854	0.467	0.997	0.831
	N6 vs. N2	0.412	1.0	0.896	0.415	0.998	0.812

## Data Availability

Data are contained within the article.

## References

[1] Liang H., Tao D., Zhang Q., Zhang S., Wang J., Liu L., Wu Z., Sun W. (2021). Nitrogen fertilizer application rate impacts eating and cooking quality of rice after storage. PLoS ONE.

[2] Zhen S., Zhou J., Deng X., Zhu G., Cao H., Wang Z., Yan Y. (2016). Metabolite profiling of the response to high-nitrogen fertilizer during grain development of bread wheat (*Triticum aestivum* L.). J. Cereal Sci..

[3] Yilan X.U., Aibin F.U., Liu T. (2019). Effects of Different Nitrogen Application Patterns on Dry Matter Accumulation and Yield of Rice in Double Cropping Rice Field. Crop Res..

[4] Alves G.H., Ferreira C.D., Vivian P.G., Monks J.L.F., Elias M.C., Vanier N.L., de Oliveira M. (2016). The revisited levels of free and bound phenolics in rice: Effects of the extraction procedure. Food Chem..

[5] Kim S.-H., Yang Y.-J., Chung I.-M. (2020). The Effect of Degree of Milling on the Nutraceutical Content in Ecofriendly and Conventional Rice (*Oryza sativa* L.). Foods.

[6] Saleh A.S.M., Wang P., Wang N., Yang L., Xiao Z. (2019). Brown Rice Versus White Rice: Nutritional Quality, Potential Health Benefits, Development of Food Products, and Preservation Technologies. Compr. Rev. Food Sci. Food Saf..

[7] Chen M., Huang W., Yin Z., Zhang W., Kong Q., Wu S., Li W., Bai Z., Fernie A.R., Huang X. (2022). Environmentally-driven metabolite and lipid variations correspond to altered bioactivities of black wolfberry fruit. Food Chem..

[8] Fàbregas N., Fernie A.R. (2019). The metabolic response to drought. J. Exp. Bot..

[9] Ti H., Li Q., Zhang R., Zhang M., Deng Y., Wei Z., Chi J., Zhang Y. (2014). Free and bound phenolic profiles and antioxidant activity of milled fractions of different indica rice varieties cultivated in southern China. Food Chem..

[10] Qiu Y., Liu Q., Beta T. (2010). Antioxidant properties of commercial wild rice and analysis of soluble and insoluble phenolic acids. Food Chem..

[11] Liu Y., Kong Z., Liu J., Zhang P., Wang Q., Huan X., Li L., Qin P. (2020). Non-targeted metabolomics of quinoa seed filling period based on liquid chromatography-mass spectrometry. Food Res. Int..

[12] Gong E.S., Luo S.J., Li T., Liu C.M., Zhang G.W., Chen J., Zeng Z.C., Liu R.H. (2017). Phytochemical profiles and antioxidant activity of brown rice varieties. Food Chem..

[13] Mbanjo E.G.N., Kretzschmar T., Jones H., Ereful N., Blanchard C., Boyd L.A., Sreenivasulu N. (2020). The Genetic Basis and Nutritional Benefits of Pigmented Rice Grain. Front. Genet..

[14] Gao Y., Guo X., Liu Y., Zhang M., Zhang R., Abbasie A.M., You L., Li T., Liu R.H. (2018). Comparative assessment of phytochemical profile, antioxidant capacity and anti-proliferative activity in different varieties of brown rice (*Oryza sativa* L.). LWT-Food Sci. Technol..

[15] Lin P.-Y., Li S.-C., Lin H.-P., Shih C.-K. (2019). Germinated brown rice combined with Lactobacillus acidophilus and Bifidobacterium animalis subsp. lactis inhibits colorectal carcinogenesis in rats. Food Sci. Nutr..

[16] Quagliariello V., Iaffaioli R., Falcone M., Ferrari G., Pataro G., Donsì F. (2016). Effect of pulsed electric fields—Assisted extraction on anti-inflammatory and cytotoxic activity of brown rice bioactive compounds. Food Res. Int..

[17] Arbona V., Manzi M., De Ollas C., Gómez-Cadenas A. (2013). Metabolomics as a Tool to Investigate Abiotic Stress Tolerance in Plants. Int. J. Mol. Sci..

[18] Shen T., Xiong Q., Zhong L., Shi X., Cao C., He H., Chen X. (2019). Analysis of main metabolisms during nitrogen deficiency and compensation in rice. Acta Physiol. Plant..

[19] Glaubitz U., Erban A., Kopka J., Hincha D.K., Zuther E. (2015). Metabolite Profiling Reveals Sensitivity-Dependent Metabolic Shifts in Rice (*Oryza sativa* L.) Cultivars under High Night Temperature Stress. Procedia Environ. Sci..

[20] Desta K.T., Hur O.S., Lee S., Yoon H., Shin M.-J., Yi J., Lee Y., Ro N.Y., Wang X., Choi Y.-M. (2022). Origin and seed coat color differently affect the concentrations of metabolites and antioxidant activities in soybean (*Glycine max* (L.) Merrill) seeds. Food Chem..

[21] Dare A.P., Grey A., Guo G., Demarais N., Cordiner S., McGhie T., Boldingh H., Hunt M., Deng C., Günther (2022). Resolving the developmental distribution patterns of polyphenols and related primary metabolites in bilberry (*Vaccinium myrtillus*) fruit. Food Chem..

[22] Campos R.I. (2001). Studies of Wound-Induced Phenylpropanoid Metabolism in Fresh-Cut Romaine Lettuce and Its Modification by Heat Shock.

[23] Liu Y., Liu J., Liu M., Liu Y., Strappe P., Sun H., Zhou Z. (2020). Comparative non-targeted metabolomic analysis reveals insights into the mechanism of rice yellowing. Food Chem..

[24] Adom K.K., Sorrells A.M.E., Liu R.H. (2005). Phytochemicals and Antioxidant Activity of Milled Fractions of Different Wheat Varieties. J. Agric. Food Chem..

[25] De Guzman M.K., Parween S., Butardo V.M., Alhambra C.M., Anacleto R., Seiler C., Bird A.R., Chow C.-P., Sreenivasulu N. (2017). Investigating glycemic potential of rice by unraveling compositional variations in mature grain and starch mobilization patterns during seed germination. Sci. Rep..

[26] Zhang H., Shao Y., Bao J., Beta T. (2015). Phenolic compounds and antioxidant properties of breeding lines between the white and black rice. Food Chem..

[27] Miller S.B., Heuberger A.L., Broeckling C.D., Jahn C.E. (2019). Non-Targeted Metabolomics Reveals Sorghum Rhizosphere-Associated Exudates are Influenced by the Belowground Interaction of Substrate and Sorghum Genotype. Int. J. Mol. Sci..

[28] Zhao C.-N., Zhang J.-J., Li Y., Meng X., Li H.-B. (2018). Microwave-Assisted Extraction of Phenolic Compounds from *Melastoma sanguineum* Fruit: Optimization and Identification. Molecules.

[29] Krasensky J., Jonak C. (2012). Drought, salt, and temperature stress-induced metabolic rearrangements and regulatory networks. J. Exp. Bot..

[30] Byeon Y.S., Hong Y.-S., Kwak H.S., Lim S.-T., Kim S.S. (2022). Metabolite profile and antioxidant potential of wheat (*Triticum aestivum* L.) during malting. Food Chem..

[31] Chung R.-S., Chen C.-C., Ng L.-T. (2010). Nitrogen fertilization affects the growth performance, betaine and polysaccharide concentrations of *Lycium barbarum*. Ind. Crops Prod..

